# Xenografting Human Musculoskeletal Sarcomas in Mice, Chick Embryo, and Zebrafish: How to Boost Translational Research

**DOI:** 10.3390/biomedicines12081921

**Published:** 2024-08-21

**Authors:** Veronica Giusti, Giacomo Miserocchi, Giulia Sbanchi, Micaela Pannella, Claudia Maria Hattinger, Marilena Cesari, Leonardo Fantoni, Ania Naila Guerrieri, Chiara Bellotti, Alessandro De Vita, Chiara Spadazzi, Davide Maria Donati, Monica Torsello, Enrico Lucarelli, Toni Ibrahim, Laura Mercatali

**Affiliations:** 1 Osteoncology, Bone and Soft Tissue Sarcomas and Innovative Therapies Unit, IRCCS Istituto Ortopedico Rizzoli, 40136 Bologna, Italy; veronica.giusti@ior.it (V.G.); giulia.sbanchi@ior.it (G.S.); micaela.pannella@ior.it (M.P.); claudia.hattinger@ior.it (C.M.H.); marilena.cesari@ior.it (M.C.); leonardo.fantoni@ior.it (L.F.); anianaila.guerrieri@ior.it (A.N.G.); chiara.bellotti@ior.it (C.B.); toni.ibrahim@ior.it (T.I.); laura.mercatali@ior.it (L.M.); 2 Preclinic and Osteoncology Unit, Biosciences Laboratory, IRCCS Istituto Romagnolo per lo Studio dei Tumori (IRST) “Dino Amadori”, 47014 Meldola, Italy; giacomo.miserocchi@irst.emr.it (G.M.); alessandro.devita@irst.emr.it (A.D.V.); chiara.spadazzi@irst.emr.it (C.S.); 3 Department of Medical and Surgical Sciences (DIMEC), University of Bologna, 40126 Bologna, Italy; 4 Orthopaedic Oncology Unit, IRCCS Istituto Ortopedico Rizzoli, 40136 Bologna, Italy; davidemaria.donati@ior.it

**Keywords:** patient-derived xenografts (PDX), cell-derived xenografts (CDX), murine models, zebrafish, chorioallantoic membrane (CAM), musculoskeletal sarcomas

## Abstract

Musculoskeletal sarcomas pose major challenges to researchers and clinicians due to their rarity and heterogeneity. Xenografting human cells or tumor fragments in rodents is a mainstay for the generation of cancer models and for the preclinical trial of novel drugs. Lately, though, technical, intrinsic and ethical concerns together with stricter regulations have significantly curbed the employment of murine patient-derived xenografts (mPDX). In alternatives to murine PDXs, researchers have focused on embryonal systems such as chorioallantoic membrane (CAM) and zebrafish embryos. These systems are time- and cost-effective hosts for tumor fragments and near-patient cells. The CAM of the chick embryo represents a unique vascularized environment to host xenografts with high engraftment rates, allowing for ease of visualization and molecular detection of metastatic cells. Thanks to the transparency of the larvae, zebrafish allow for the tracking of tumor development and metastatization, enabling high-throughput drug screening. This review will focus on xenograft models of musculoskeletal sarcomas to highlight the intrinsic and technically distinctive features of the different hosts, and how they can be exploited to elucidate biological mechanisms beneath the different phases of the tumor’s natural history and in drug development. Ultimately, the review suggests the combination of different models as an advantageous approach to boost basic and translational research.

## 1. Introduction

Sarcomas account for less than 1% of adult malignancies and 12–15% of paediatric tumors [1]. Historically, sarcomas have been categorised according to the anatomical site of occurrence in Bone Sarcomas (BS) (15%), Soft Tissue Sarcomas (STS) (80%) and Gastro-Intestinal Stromal Tumors (GIST) (5%) [2]. Nowadays, the World Health Organization distinguishes over 100 histological subtypes with peculiar morphology and molecular traits [3,4]. Moreover, many histotypes are exceedingly rare (less than 1 case/1,000,000 persons) [5]. More than 90% of BS are classified as osteosarcomas (OS), Ewing sarcomas (EWS), or chondrosarcomas (CHS) [3]. Among STS, liposarcoma (LPS) and leiomyosarcoma (LMS) represent the most common histologic subtypes in adults, while rhabdomyosarcoma (RMS) is the most prevalent histotype in children [6]. The reader is referred to Table 1 for clinicopathological details. To date, the gold standard treatment for localised sarcoma is surgery, often in combination with radio- and/or polychemotherapy [1]. Unfortunately, around one third of sarcoma patients still have a dismal prognosis, and those with metastatic disease medially survive only 12 months [2]. Targeted drugs may overcome the limits of current therapies, however, the selection of proper actionable targets in sarcomas is challenging [1]. Based on omic analyses, a plethora of novel therapies has been proposed, however, their translation into the clinical praxis is discouraged by the unsatisfactory results obtained in clinical studies so far [7,8]. This reflects the lack of faithful research tools to model cancer phenotype at the molecular and organism levels [1,7]. Therefore, preclinical models that incorporate the biology and genetics of human cancers and preserve inter- and intra-tumor heterogeneity are compelling to obtain reliable and translatable results [7,8]. On the other hand, the drug-screening pipeline requires high-throughput and time-effective systems. Cell line-derived xenograft (CDXs) models have represented, up to now, the workhorse for basic and translational research, thanks to their consistency and cost-effectiveness, but patient-derived xenografts (PDXs) are currently recognized as the most effective preclinical model for phenocopying cancer biology and architecture, and studying drug response and resistance [8,9]. Both technical (low success rate and through-put, long experimental time, lack of tools to follow tumor growth) and intrinsic (lack of immune milieu, stroma substitution by host cells [10], different pharmacokinetics, and long-term model instability [11,12]) concerns profoundly burden this model [7]. The transposition of the EU Directive 2010/63/EU on the protection of animals used for scientific purposes under national laws has resulted in strict regulation, reflecting the growing ethical concerns around the use of adult animal experimentation [13]. Therefore, researchers are turning their attention to non-mammalian embryonal models, such as zebrafish larvae [14] and chick embryos [15]. Embryonal models fall under the aegis of less restrictive regulation [16] until they are capable of independent feeding (chick embryo: hatching; zebrafish: 5 days post-fertilization (dpf) [17]) and, most importantly, until they acquire the ability to feel pain (chick embryo: >13 egg development day (EDD) [18,19]; zebrafish: 5 dpf). Moreover, mice are kept under conditions that respect animal welfare, but do not represent life “as in the wild”—on the contrary, embryonal systems do not suffer environmental distress. Technically speaking, embryonal systems hold the promise of higher manageability without the need for animal facilities, higher throughput, and lower costs [14,15,20]. Zebrafish (ZF) has emerged as an innovative in vivo model due to its unique combination of genetic and physiological features. Its rapid development, embryo body transparency, high fecundity and easy genetic manipulation make ZF a suitable approach for preclinical studies [14]. Moreover, the ZF genome has been completely sequenced showing high homology to human DNA, and the larvae are therefore recognized as a suitable organism for genetic manipulation [21]. In this scenario, ZF xenografts represent a unique approach to investigate complex biological processes, to uncover molecular mechanisms, and to develop drug-screening platforms [14,22]. The chicken chorioallantoic membrane (CAM) model has been widely used for studies on angiogenesis and tumorigenesis on prostate cancer, glioblastoma, OS, and lung adenocarcinoma, thanks to its transparency and strongly vascularized structure [23]. Moreover, the CAM can host xenografts of human tumor cells and patient-derived fragments, given its immature immune system [24]. CAM has many advantages, such as ease of access, short experimental times, and cost-effectiveness. Xenografting human tumor cells or tissues on CAM induces angiogenesis, and this can be exploited for both basic research and the development of novel antiangiogenic therapies [25]. The choice of the host and of the procedure for a xenograft strictly depends on the experimental question of interest. The intrinsic and technical peculiarities of the different models can be exploited to represent and elucidate biological mechanisms which are specific to the different phases of the tumor’s natural history. This review of musculoskeletal sarcomas (MSKT) models aims to highlight the peculiar features and potentialities of the different xenograft hosts in the study of the different steps of tumor natural history and drug development, with the aim of generating awareness of the choice of the proper model and to prompt the integration of different models, boosting basic and translational research.

**Table 1:** Sarcomas of the musculoskeletal system. The table reports the main molecular and clinical features of selected MSKT.

Sarcoma	Type	Pathognomonic Feature	Incidence ^@^	5-Year Overall Survival	Primary Site	Main Metastasis Site	Therapy	Refs.
Osteosarcoma (OS)	BS	Multiple chromosomal aberrations; frequent TP53 and RB1 mutations; DNA helicase disorders	3–4.5 *	60% *	Knee, humerus	Lungs	Neo-adjuvant and adjuvant chemotherapy (Methotrexate, Adriamycin, Cisplatin (MAP)); surgery or radiotherapy where surgery is not possible	[26,27]
Ewing sarcoma (EWS)	BS	85–90% EWS::FLI1 translocation	<1	70% ^#^	Long and flat bones	Lungs, bone, bone marrow	Induction chemotherapy (doxorubicin, etoposide, cyclophosphamide, vincristine, and ifosfamide); surgery; radiotherapy	[28]
Chondrosarcoma (CHS)	BS	IDH1/2, EXT1/2 mutations	<6	>90% (low grade); 75% (II grade); 30% (III grade)	Proximal femur, humerus, tibia, pelvis and scapula	Lungs	Surgery	[29,30,31]
Liposarcoma (LPS)	STS	MDM2, CDK4 amplification	10	93% (grade I ^§^); 57% (grade II ^§§^); 21% (grade III ^§§^)	Most commonly in thigh, retroperitoneum, inguinal region and popliteal fossa	Retroperitoneum, distant sites and soft tissue sites ^§§^	Surgery; chemoradiotherapy	[32,33]
Rhabdo-myosarcoma (RMS)	STS	PAX3::FOXOA1 and PAX7::FOXOA1 translocations; RAS-PI3K, RTK signaling, loss of PTEN, TP53 and CDKN2A in fusion negative RMS	4.5 ^^^	>70%	Head, neck, genitourinary tract, limbs	Lungs, bone and bone marrow	Surgery, ionizing radiation, chemotherapy (vincristine, actinomycin D, cyclophosphamide (VAC); Ifosfamide)	[34]
Myxofibrosarcoma (MFS)	STS	Highly complex karyotypes	1	75%	Lower limbs, trunk, head and neck	Lungs, bone and lymph nodes	Surgery; radiotherapy; chemotherapy (doxorubicin, ifosfamide)	[35,36]
Synovial Sarcoma (SS)	STS	95% SYT::SSX1/2/4 translocation	1–2	76% ^#^	Extremities	Lungs, bone and lymph nodes	Surgery; radiotherapy; chemotherapy (anthracycline *plus* ifosfamide, gemcitabine, docetaxel, trabectidin, VAC)	[37]
Leiomyosarcoma (LMS)	STS	Highly complex karyotypes with genomic instability	6	50%	Commonly in peritoneum and extremities	Lungs, peritoneum, liver, and bone	Surgery; radiotherapy; chemotherapy (doxorubicin)	[33]
Undifferentiated pleomorphic sarcoma (UPS)	STS	Highly complex karyotypes	8–10	48%	Long bones, preference for proximal tibia and distal femur	Lungs	Surgery; radiotherapy; chemotherapy (anthracycline and ifosfamide)	[38,39]

Notes: @ cases/10^6^ population/year; * in children and adolescents; ^#^ local disease; ^§^ well-differentiated LPS; ^§§^ de-differentiated LPS; ^^^ aged under 20.

## 2. Xenografting MSKT

**Figure 1:**
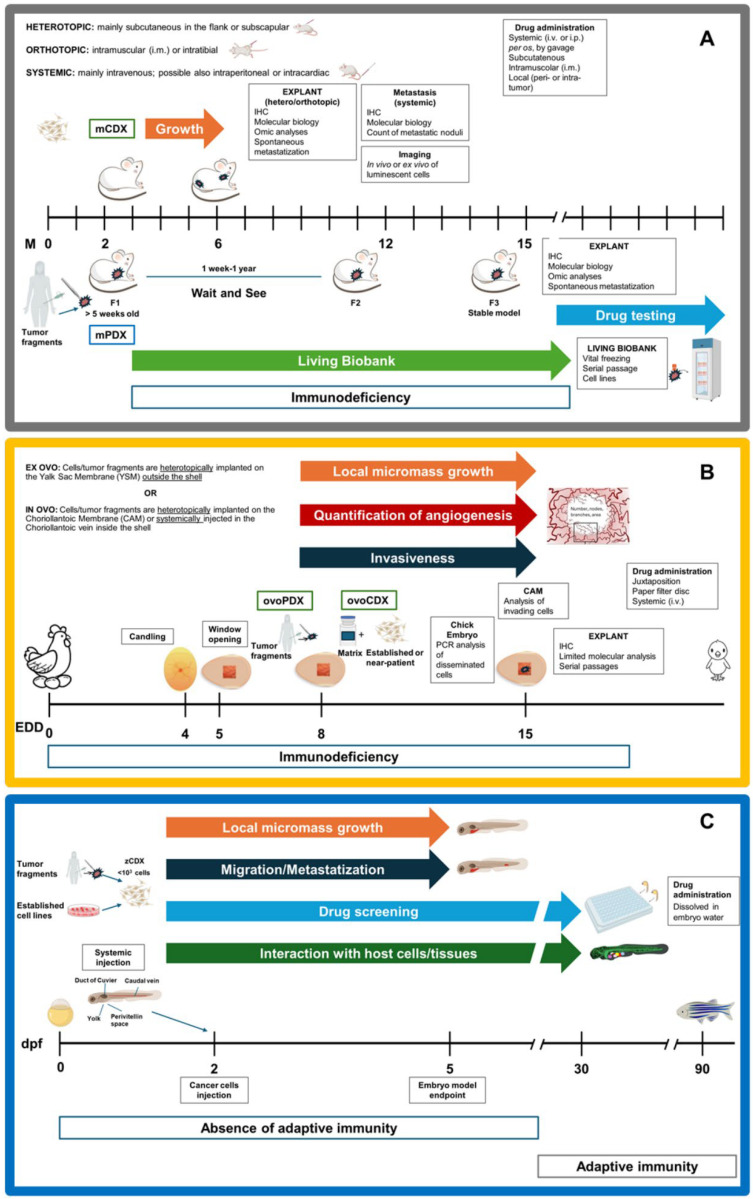
Methodologies and applications of CDXs and PDXs in the different hosts. Each figure reports a timeline of xenografting experiment, including technical details and downstream applications. (**A**) mice; (**B**) chick embryo; (**C**) zebrafish larvae. Timeline specifications: M: months; EDD: egg development days; dpf: days post-fertilization.

**Figure 2:**
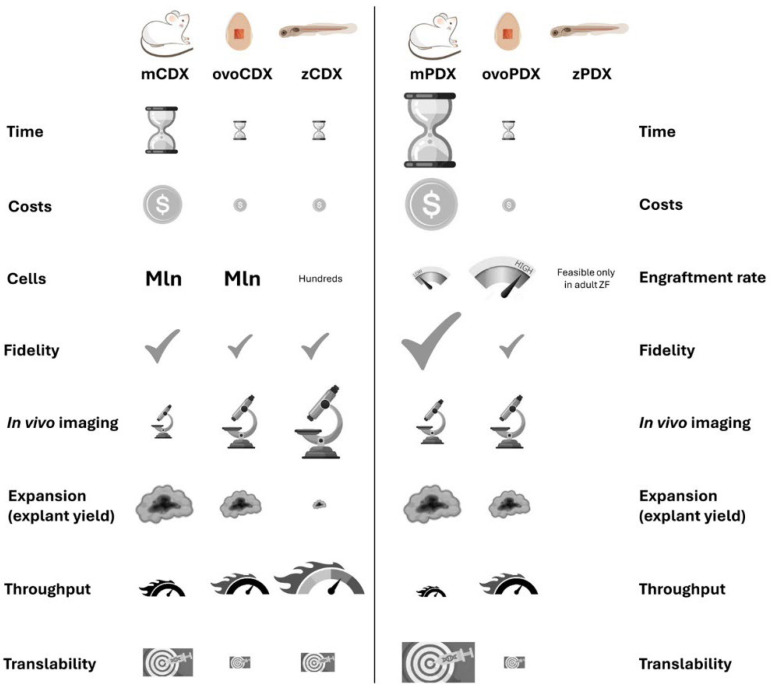
Comparison of CDXs and PDXs in the different hosts. The table highlights the limits and potentialities of xenograft in mouse, chick embryo, and zebrafish larvae.

According to the National Cancer Institute Dictionary of Cancer Terms, the term “xenograft” refers to the transplant of an organ, tissue, or cells to an individual of another species [40]. While xenografts obtained by injection of established or near-patient cells (cell-derived xenografts, CDXs) are more reproducible and provide higher throughput, xenografting tumor fragments (patient-derived xenografts, PDXs) has the advantage of maintaining cellular heterogeneity and architectural features of the original tissue [8]. Xenografts of MSKT have been reported in rodents, especially in mice (m), embryonic and adult ZF (z), and CAM (ovo). Notably, xenografts in mice are regulated by the EU Directive on the protection of animals used for scientific purposes, while embryonal models are not considered animals until they are able to feed themselves, hence simplifying bureaucracy [16]. In the following paragraphs, technical details of the xenografting procedure in the different hosts are discussed for the sake of comparison (Figure 1 and Figure 2). The description of the detailed protocols for the obtainment of CDXs and PDXs from each host is beyond the scope of this review, therefore, the readers are referred to technical papers and reviews for further information [41,42,43,44,45,46,47,48].

The earliest reports of xenotransplantation of MSKT into nude mice were published around the 1980s [49,50,51], but mPDX have re-gained momentum only in the last two decades. Obtaining an mPDX is a demanding endeavour, requiring an adequate tumor specimen (>1 mm^3^ and 10% viability [52]) and immune-compromised mice, which entail cumbersome sterile handling, significant costs and time (latency reaches 1 year [53]), imaging systems adapted to animal hosts, and experience in handling, given the low engraftment rate (Table 2). Moreover, an mPDX should be serially transplanted at least twice to obtain a stable model, and validated for its fidelity to the original tumor (Figure 1A) [7]. Indeed, preserving the tumor microenvironment from the donor tumor is the premise for the study of the tumor behaviour in vivo. However, passage after passage, the tumor stroma is increasingly replaced by the host stroma, and a decrease in tumor fidelity is generally observed. The replacement of the stroma is mostly considered an acceptable drawback of the model, since Arnaud Blomme et al. demonstrated that the metabolic profiles of both tumor cells and stromal cells remain stable for at least four passages, even though the replacement began at the second passage [10]. Ultimately, this large amount of work is rewarded by the creation of living biobanks of MSKT, which virtually eternalize a tumor specimen and render it available for future studies (Table 2). In respect to mPDX, establishing ovoPDXs is simpler to accomplish. Back in 1911, CAM represented the very first host for xenografts, while MSKT were transplanted only in 2012 [44,54]. Indeed, the high density of blood vessels creates an ideal milieu for tumor growth due to the ubiquitous supply of oxygen, nutrients, and growth factors [55]. In addition, ovoPDXs present unique advantages as grafts are visible to the naked eye, allowing for easy monitoring under a stereomicroscope [15]. Chick embryos are inexpensive and easy to manipulate as they are immunodeficient up to 18 EDD, experiments run in 10 days, and engraftment rates reach 80% in MSKT [44]. However, this ease comes at a price. Indeed, the amount of tumor tissue retrievable at the end of the experiment is similar to that at the start, limiting the possibility of down-stream molecular analysis (Figure 1B). For this reason, even if serial passages of ovoPDX are feasible up to 8 times in OS [56], these models cannot contribute to the creation of living biobanks. Notably, ovoPDXs allow for the mimicking of neoangiogenesis in vivo: indeed, after a first avascular phase of around 72–96 h after implantation, neovascularization occurs, in which the anastomosis between the CAM vasculature and the tumor vasculature is established, providing nutrients for a rapid growing phase [57]. Finally, one research group reported the feasibility of zPDX of gastrointestinal tumors in embryos [58,59] and in adult fish [60], however, the application of this technique is discouraged by the small embryo size.

CDXs can be obtained from established or near-patient cells, including primary and mPDX-derived cells. mCDX with established cell lines and their gene-edited or drug-resistant variants have been considered the workhorse for basic and translational research [9]. They usually require 1–5 × 10^6^ cells/mouse and experiments run over a few months (Table 3 and Table 4; Figure 1A). mCDX of near-patient cells represent a win–win approach that couples the fidelity and heterogeneity of patient-derived samples with the manageability, time-effectiveness, and scalability of CDX [61]. Even though CAM is an established in vivo model to evaluate the progression of CDXs [15], there are only a few reports describing its use for BS and STS analysis, mostly focused on OS. In 2010, Balke et al. reported the ability of different OS cell lines (MNNG-HOS, U2OS and SaOS) to form vascularized solid tumors on CAM after four days of incubation [25]. Of note, the amount of xenografted cells for ovoCDX may be equal or even superior to mCDX, but experimental times are shorter (Figure 1B). On the other hand, zCDX can be considered miniature models as they only require <10^3^ cells, rendering them particularly suitable for precious near-patient cells [62] (Figure 1C). zCDX also minimises experimental timelines to just a few days, as well as costs and the manipulation. Moreover, ZF embryos stand out for their optical transparency, facilitating in vivo imaging of engrafted cells [14].

The morphological and morphometric evaluation of newly formed tissues is feasible in all xenograft hosts, but it is more challenging in ZF samples due to their small size and the limited number of available antiZF antibodies [63]. Down-stream molecular analysis is theoretically feasible for all the models, yet the amount of the available material is limited in the case of ZF and chick embryo. Technological development, especially in the field of imaging, has opened up new possibilities to quantify primary tumor growth and the study of tumor cells’ migration in the embryonal models.

In the next sections, we will compare different xenograft hosts and modalities, and highlight their peculiarities with respect to the different steps of tumor natural history, i.e., tumorigenesis, angiogenesis, and metastatization, as well as their potentialities in translational research.

**Table 2:** mPDX collections for MSKT. The table reports injection site, engraftment rate, latency of selected collections of MSKT mPDX, and their applications.

Tumor-Related Features		PDX-Related Features			Applications	Refs.
Sarcoma Histotype	Inoculated Mass	Injection Site	Tumor Take (%)	Latency of 1° Passage		
OS and EWS	nd	flank, s.c.	25/49 (51%)	16–280 days	modeling	[64]
LPS, LMS, MFS, SS, UPS, CHS and other ^$^	25–75 mm^3^	bilaterally s.c.	32/188 (17%)	ns	drug testing; Xenosarc platform	[65,66]
CHS	2 mm^3^	s.c.	ns	ns	omic analysis	[67]
OS	2–3 mm^3^	flank, s.c.	15/37 (40.5%)	19–225 days	modeling; clinicopathological correlations	[52]
OS and EWS	4 mm^3^	transscapular brown fat, s.c.	OS:22/61 (36%) EW:7/29 (24%)	1 week–1 year	modeling; target dependencies; drug testing	[53]
LPS	4 × 4 mm	bilaterally s.c.	7/10 (70%)	2–9 months	drug testing	[68,69]
OS, EWS, RMS	2 × 2 × 1 mm	s.c.	EW: 17/41 (41.55%); OS: 5/12 (41.7%); RMS: 8/15 (53.3%)	ns	modeling; clinicopathological correlations; drug testing	[70]
OS, RMS	2 × 2 mm^3^	flank, s.c.	OS: 51.4% RMS: 53.8%	300 days	target dependencies	[71]
OS	3 mm^3^	flank, s.c.	9/21 (42.9%)	3–9 weeks	modeling	[72]
LPS	diameter of 3–5 mm	lower back	25/56 (44.64%)	ns	drug testing	[73]
OS	2 mm in diameter	flank, s.c.	20/57 (35.1%)	ns	modeling; clinicopathological correlations	[74]
LMS	ns	s.c.	17/49 (35%)	ns	target dependencis	[75]
OS, EWS, RMS, SS, other *	2–5 mm^3^	interscapular fat pad, or i.m.	76/131 (58%)	12–285 days	modeling; target dependencies; drug testing; MAPPYACTS	[76]
Sarcoma (NCI PDXNet consortium)	ns	s.c.	ns	ns	modeling; clinicopathological correlations; target dependencies	[77]
RMS	25–30 mm^3^	flank, s.c.	50% (6/12)	195 days (median)	modeling	[78]
OS, LMS, LPS	1–2 mm^3^	flank, s.c.	ns	ns	modeling; EuroBoNeT	[79,80]
BS	3 × 3 × 3 mm	flank, s.c.	16	19–125 days	modeling	[63]
STS	5-mm fragments	orthotopic, i.m. or s.c.	32/107 (29.9%)	9–184 days	modeling; clinicopathological correlations	[81]
OS, EWS, RMS, other ^§^	single-cell suspension	orthotopic	OS 15/31 (48%); RMS 13/20 (65%); EWS 2/7 (29%); other 5/6 (83%)	1–11 months	modeling; drug screening on primary colture; drug testing	[82]
OS, EWS, LPS, UPS, SS other	2 mm^3^	s.c.	7/12 (58%)	ns	target dependencies and drug identification	[83]

Notes: ns: not specified; * also includes solid tumors, CNS, lymphoma, and leukemia; ^$^ atypical lipomatous tumor, undifferentiated sarcoma not otherwise specified, solitary fibrous tumor, undifferentiated spindle cell sarcoma, extra-skeletal myxoid chondrosarcoma *plus* ultra-rarer histotypes, BCOR/CIC; ^§^ desmoid small round cell tumor; i.m.: intramuscular; s.c.: subcutaneous.

**Table 3:** mCDX for the evaluation of oncogenes and oncosuppressors. For each paper, the table describes the main features of xenografted cells and experimental details of selected mCDX determining the role of genes in tumorigenesis.

Sarcoma	Cells			Mice			Gene		Refs.
MSKT	ID	Type	Quantity (×10^6^)	Injection Site	Mice/Experimental Group	Experimental Time (Days)	ID	Role	
OS	143.98.2 OS	Est.	1	Heterotopic	6	25	STAT3	↑	[84]
OS	143B PML BK TK	Est.	10	Heterotopic	5	nd	CHEK1	↑	[85]
OS	MG63	Est.	1	Heterotopic	nd	30	GFRA1	↑	[86]
OS	MNNG-HOS	Est.	1.5	Heterotopic	5	20	S1PR3	↑	[87]
OS	MNNG-HOS	Est.	5	Heterotopic	20	21	FGD1	↑	[88]
OS	MNNG-HOS	Est.	2	Heterotopic/Orthotopic	≤6	25 *	KIF18B	↑	[89]
OS	U2OS	Est.	1	Heterotopic	10	28	NG6X	↓	[90]
OS	143B	Est.	0.5	Orthotopic	5	30	FOXP1	↑	[91]
OS	pOS-1	Near-pt.	5	Heterotopic	6	42	Gαi3	↑	[92]
OS	pOS-1	Near-pt.	4	Orthotopic	na	28	TIMM13	↑	[93]
OS	K-HOS	Est.	1	Heterotopic	7	≥35	RIPK1	↑	[94]
EWS	RDES, TC-32	Est.	3/0.2	Heterotopic	≤6	nd	SOX6	↑	[95]
EWS	A673	Est.	2	Heterotopic	6	25	KLF15 or TCF4	↑	[96]
EWS	A4573	Est.	4	Heterotopic	10	20	CAV1	↑	[97]
EWS	TC-71	Est.	1	Heterotopic	7	50	RIPK1	↑	[94]
EWS	A673	Est.	2	Heterotopic	5	>60	miR34a	↓	[98]
RMS	RD	Est.	1.25/1.5	Heterotopic	≤7	45	HES1-YAP1-CDKN1C	↑	[99]
RMS	Rh30, Rh41	Est.	0.75–1	Heterotopic	40	40/75	miR-486-5p	↓	[100]
RMS	Rh18, Rh30, C265S-Rh30 mutant	Est.	2	Orthotopic	≤10	≤45	PANX1	↓	[101]
LPS	DDLPS 246	Near-pt.	1–2	Heterotopic	6	45	miR-133a	↓	[102]

Notes: Est: established; near-pt: near-patient; nd: not disclosed; na: not available ↑ oncogene; ↓ oncosuppressor; * time to amputation.

**Table 4:** mCDX for the evaluation of metastatic potential. For each paper, the table describes the main features of xenografted cells and experimental details of selected mCDX determining the role of genes in metastatic potential.

Sarcoma	Cells			Mice				Gene		Refs.
MSKT	ID	Type	Quantity (×10^6^)	Injection Route	Metastasis Site	Mice/Experimental Group	Experiment Time (Days)	ID	Role	
OS	143B, MG63.2	Est.	1.5	Orthotopic	Lung	5	28, 56	IGFBP5	↓	[103]
OS	143B	Est.	0.5	Orthotopic	Lung	≥6	20	CXCR4	↑	[104]
OS	SaOS-LM7	Est. *	1	Systemic	Lung	10	49/56	BMP-2	nd	[105]
OS	143B	Est., drug-resistant	0.5	Orthotopic	Lung	8	40	MIG-7	↑	[106]
OS	U2OS	Est.	10	Heterotopic	Lung	6	70	ROCK2	↑	[107]
OS	MG-63/Akaluc	Est.	1	Systemic	Lung	≤8	nd	LPAR1	↑	[108]
OS	MG63, SaOS2	Est.	1	Systemic	Lung	11	21	TRIM7	↑	[109]
EWS	TC71	Est.	0.5	Metastatic site (chestwall)	Lung	9	nd	PDGFR-β	↑	[110]
EWS	A673	Est.	2	Systemic	Lung and lymph nodes	6	98	IGF2BP3	↑	[111]
EWS	TC71, EWS4	Est., near-pt	0.01	Systemic	Lung, kidneys, retroperitoneum	5, 10	150, 250	PORCN	↑	[112]
EWS	A673 Luc	Est.	0.25	Orthotopic	Lung and bone	≤15	28	ZYX/ITGA5	↓	[113]

Notes: Est: established; near-pt: near-patient; nd: not disclosed; ↑ oncogene; ↓ oncosuppressor; * established by 7 cycling of parental cell line through the lungs of nude mice.

## 3. Tumorigenesis

A better understanding of the initial molecular events that lead to an irreversibly transformed cellular state might revolutionise early detection strategies as well as therapeutic success rates [114]. The models of election for the study of the formation of tumors are represented by carcinogen exposure-induced tumors, genetically engineered mouse models (GEMMs) [115,116,117], and gene-edited ZF [118,119,120]. However, these models are out of the scope of this review and the reader is referred to the cited literature for MSKT de novo models. Regardless of the host, PDX are not ideal models to study tumorigenesis as they intrinsically consist of the injection of a preformed tumor in a living organism. However, the transplanted tumors can be gene-edited in vivo to determine the contribution of a specific oncogene to tumor growth: for example, OS mPDXs were administered adenovirus-associated virus (AAV) carrying shFOXP1 to confirm its oncogenic role in OS [91]. Similarly, the onco-suppressive role of miR193b was verified in vivo, transfecting established mPDX of dedifferentiated LPS with the miRNA itself or a small molecule inhibiting its target [121]. Other than this approach, mPDXs are correlatively exploited if they express and/or lack the gene of interest. mPDXs with high basal expression of TAp73 or activated Hedgehog pathways were used to confirm the pro-tumorigenic role of PLK2 [122] and Gli/Smo [123] and the efficacy of their pharmacological inhibition in OS. Additionally, the pro-tumorigenic role of SOX6, demonstrated via gene-editing approaches, was eventually validated in a collection of mPDX of EWS, where SOX6 levels correlated with a higher Ki67 index [95]. Gene-edited cells have been the workhorses for studying the impact of genes on tumorigenesis for years, both in vitro and in vivo. This approach, together with the use of specific inhibitors, has elucidated the contribution of oncogenes, oncosuppressors, miRNAs [98,100,124], and related pathways in sarcomagenesis (Table 3). Notably, Slemmons and colleagues generated and xenografted YHR and YHV cells harbouring multiple transgenes to elucidate the role of YAP in RMS [125]. Other researchers have, instead, exploited inducible models to study the interaction of other oncogenes with EWS::FLI1 [96]. To circumvent the limitations in the gene-editing of near-patient cells, mice bearing OS primary-CDX were infected with AAV carrying shGαi3 [92], with a similar approach to that used on mPDX. Finally, the oncogenic role of Gαi3 [92] and TIMM13 [93] in OS was corroborated via mCDX of CrispR/Cas9-edited near-patient cells. mCDXs of gene-edited cells require the obtainment of stable-modified models, which is time-consuming and may introduce variability due to sub-cloning and inconsistencies with in vitro experiments. The use of fast-developing embryonal models allows us to employ the same transient strategies (siRNA, antisense, miRNA antagonists, soluble molecules, and transfections) used in vitro in the in vivo setting, combining time-effectiveness and consistency in methodology and robustness of results. This approach was exploited to evaluate the role of the activation of Akt in RMS, using the same plasmid for myrAkt, both in vitro and in ZF, completely avoiding the use of mice [126]. A similar approach in ovoCDX permit the uncovering of the reliance of SS on YAP/TAZ-TEAD-mediated transcriptional activity and confirmed the efficacy of its inhibition via verteporfin [127].

## 4. Angiogenesis

Angiogenesis is essential for tumors’ growth beyond the size of 1 cm^3^, as this is an empirical limit beyond which nutrient deficiency and hypoxia occur [128]. Understanding how cancer cells hijack the natural process of angiogenesis to their favour could highlight novel therapeutic targets and help restrain tumor progression ab initio [129,130]. Angiogenesis is a very complex, spatially dynamic, and temporarily determined phenomenon, which is very difficult to dissect and visualize in an adult organism. Indeed, the qualitative evaluation and quantification of angiogenesis in vivo requires sophisticated techniques, such as injection of fluorescent and non-fluorescent markers [131], Magnetic Resonance Imaging (MRI), Positron Emission Tomography (PET), or Optical Doppler Tomography [128,132]. An alternative is represented by the in vivo angiogenesis assay (DIVAA), consisting of subcutaneous implantation of semi-closed silicone cylinders (angioreactors) into nude mice. This system was used, in combination with mCDXs, to highlight the role of microvesicular cargo in OS angiogenesis [133]. The role of EWS::FLI1 in hijacking angiogenesis and the recruitment of bone marrow-derived cells was described in mCDX of EWS cells [134,135]. mPDX are employed to evaluate the efficacy of anti-angiogenic drugs on tumor growth, rather than to study the angiogenetic mechanism per se. As one of the few examples, EYA3-targeting monoclonal antibody-drug conjugates and benzarone were tested in EWS [136].

Embryonal models, on the other hand, offer the advantage of incomparable ease of visualization and imaging. The CAM is the system of election for the study of angiogenesis, thanks to its highly vascularized bed. Xenografted cells, for example SaOS2, can induce sprouting angiogenesis, irrespective of the number of seeded cells, although the degree of the angiogenic response significantly correlates with cell number [25]. Analogously, xenografted tumor fragments also induce angiogenesis around 72–96 h after implantation and anastomose to the CAM vessels [57]. The CAM assay enables a straightforward observation of angiogenesis to the naked eye, which is not possible with other animal in vivo models. Moreover, tumor angiogenesis and vasculogenesis can further be visualized using in vivo microscopy in the CAM adjacent to the tumor [137]. Additionally, acquired images can be manually and/or semi-automatically analysed through specific software (ImageJ, AngioQuant, AngioTool) to gather quantitative information, which is of utmost interest for the screening of antiangiogenic drugs [137,138]. The study of angiogenesis in the CAM often proceeds stepwise, including chick aortic ring sprouting assays in vitro, CAM and/or yalk sac membrane (YSM) assay in ovo or ex ovo, and disease-related evaluation based on ovoCDX: such an approach has highlighted the anti-angiogenic properties of reversine in OS [139] and the various peptides in fibrosarcoma [140]. The angiogenic properties of cytokines, chemokines, growth factors, chemotherapeutics, and targeted drugs can be assessed by the direct placing of the substance of interest on the naked membranes of the chick embryo. Vimalraj and colleagues proposed a variant of YSM assay, which is classified as an in vitro model, where cells were not actually xenografted but grown on coverslips, which were simply juxtaposed on YSM. This approach highlighted the role of miR-432-5p and miR-424-5p in regulating angiogenesis in OS [141,142]. Interestingly, the data on the inhibitory effect of miR-432-5p on angiogenesis obtained in ovo were confirmed in the zCDX of OS cells previously transfected with the miRNA mimic [141].

ZF embryos represent an ideal model to study the interplay between tumor and endothelial cells [143]. Their transparency allows the monitoring of blood vessel development to evaluate the effects induced by the presence of cancer cells. The sub-intestinal vein (SIV) is a major vessel plexus in proximity to the yolk sack, commonly used to quantify the angiogenic potential through the measure of vessels spreading in surrounding tissues [144]. This model can be applied to study the angiogenic alterations induced by cancer cells or the preclinical effect of anti-angiogenic drugs. The generation of ZF strains with fluorescent vessels, together with the ease of high-content imaging, also represent great opportunities offered by this host (reviewed in [145]), which are quite underexploited in MSKT.

## 5. Metastatization

Metastatization is a multistep process, including local invasion, intravasation, and dissemination via hematogenous or lymphatic vessels, extravasation, homing, dormancy and final outgrowth of a new tumor [146]. As metastases represent the main cause of cancer-related death [147], representative metastasis models are pivotal to dissecting the molecular mechanism underlying each step and highlighting the related target dependencies [148].

Spontaneous metastasis from heterotopically implanted sarcoma mPDX represent rare events (Table 1) [53]. On the other hand, orthotopic mPDXs or mCDXs do lead to the formation of distant metastases in a wide range of target organs, other than lung and lymph nodes [149,150,151], probably due to the interaction of tumor cells with the native tumor-niche. However, metastatization takes such a long time that orthotopic primary tumors, especially in the limbs, often need to be excised and mice followed-up until metastases appear, however, this surgery represents a major ethical concern. Spontaneous metastasis models from orthotopic mPDX or mCDX were reported for EWS [152], OS [150,153,154], and UPS [155]. Interestingly, this technique represents the best setting for the preclinical evaluation of anti-metastatic drugs and was exploited in the mPDX of OS treated with a monoclonal antibody against the Wnt-signalling inhibitor dickkopf-1 [156], a β-catenin/transducin β-like protein 1 (TBL1) inhibitor alone and in combination with doxorubicin [157] and in mPDX of the Giant Cell Tumor of Bone (GCTB) treated with a monoclonal antibody targeting CD44 [158]. Established or rarely near-patient [159] mCDXs via systemic injection, which is performed mainly intravenously, lead to the formation of experimental metastasis. This route of administration eliminates the first steps of metastatization and directly disseminates cells into the bloodstream, enhancing replicability and reducing experimental times. The main metastatic site in these models is represented by the lungs, being the first organs reached by venous circulation. Systemic, orthotopic, and rarely heterotopic, mCDX served to elucidate the contribution of several genes and pathways to metastasization (Table 4). Notably, multiple mCDX with gene-edited cells were used to elucidate the pro-metastatic role of Rab22a-NeoF1 in OS and to dissect its molecular mechanism [160]. Moreover, orthotopic spontaneous and systemic experimental metastasis models have also been employed to assess the anti-metastatic efficacy of CSF1R [161], HDAC [100], PDPN [162], CDK [159], and LPAR1 [108] inhibitors in OS.

In contrast, embryonal models can be effective systems for studying the first steps of metastases (i.e., local invasion, intra- and extravasation, and dissemination). In this case, the formation of full-blown metastasis is not observable, due to the short-time experiments and to the lack of target organs. Nevertheless, it was previously demonstrated that cells systemically injected into the chick embryo do lead to tumor formation in the hatched chicks [163]. CAM assays allow the easy monitoring of the intravasation of tumor cells into the microvasculature and the determination of the invasive phenotype of ovoCDX and ovoPDX, heterotopically deposited on the CAM surface or of the experimental metastatic potential after intravenous injection in the chorioallantoic vein [41,138,164]. Metastatic cells can be distinguished from the chicken ones and quantified by IHC or qPCR of ALU sequences from the DNA of isolated chicken embryo organs [165]. Imaging of metastatic cells is feasible via CT, MRI for magnetically labeled cells, and video-microscopy or fluorescent imaging under ex ovo conditions. In the context of sarcomas, Snail, a master gene of epithelial-to-mesenchymal transition (EMT), was modulated in OS cells, which were heterotopically injected on the CAM: cells overexpressing Snail showed a higher ability to invade the stroma, and enter and migrate along the vasculature of the CAM [166]. Analogously, the pro-migratory role of IL6 was demonstrated ex ovo upon injection of EWS cells and paracrine administration of the soluble interleukin [167]. Authors have demonstrated the higher invasiveness of treated cells by visualising migrated cells under a fluorescent microscope and categorising them according to the distance from the tumor edge [167]. Moreover, the analysis of ALU sequences has revealed a greater invasion ability to the lung and liver of FUS-CHOP-transfected LPS cells [168]. No less important, the CAM system may represent a complementary assay for anti-metastatic drug testing [138,164], but no examples are available for sarcomas.

Moving attention to the ZF model, the most attractive feature is the absence of pigmentation, which makes it particularly suitable for the imaging analysis of inner organs and tissues [169]. Moreover, researchers have generated several transgenic strains expressing fluorescent proteins in specific cell populations and organs, or upon pathway activations [170]. This, together with the injection of cancer cells marked with fluorescent trackers, allows the monitoring of single-cell behaviour and the dynamic interactions with surrounding and distant tissues. High-resolution imaging technologies, such as light sheet and confocal microscopy, represent the best approach to visualize the cross-talk between endothelial and cancer cells at a single-cell level—all outputs that are difficult to reach with other animal models [9]. Through this model, Van der Ent and colleagues first demonstrated the ability of EWS cells to proliferate, migrate, and disseminate via the hematogenous route to fins, head, and body. Afterwards they exploited an Albino Casper, *TG(fli1:EGFP)* strain, characterised by fluorescent vessels, to track the amount, size, and migratory routes of tumor cell foci for each embryo upon treatment with nutlin-3a and/or YK-4-279 [171]. Using a similar approach, fluorescent RON-deficient and proficient cells were injected into a ZF line with red fluorescently tracked endothelium: RON deficiency reduced local tumor burden at injection sites and perivascular areas with metastatic cells [172]. Lastly, mCherry-labelled EWS cells were injected into the yolk sac of *fli1:EGFP* ZF embryos to verify that the inhibition of SIRT1/2 via tenovin-6 halted the invasiveness of tumor cells by tracking the distance and direction of cell clusters inside the embryo in vivo [173].

## 6. Drug Development

Modeling sarcomas is essential for designing and validating novel drugs based on the identification of specific target dependencies. mPDXs allow the amplification of patient-derived tumor samples and the faithful preservation of histologic, genomic, epigenetic, genetic, metabolic, proteomic, and clonal traits [7,9] (Table 2). Several collections are available at local laboratories (Table 2), distributors, and within European programmes (listed in [174]), providing a virtually unlimited repository of clinically annotated and molecularly characterized human samples. The integration of different omic data via algorithms, mathematical modeling, or, lately, AI-systems, can provide even more informative and comprehensive results. RNA sequencing combined with drug prediction algorithms has been employed to rank therapies in RMS tumors and related PDX: notably, in this study, all the selected drugs were evaluated in mPDX [175]. Pandyia and colleagues set up a multi-OMICS analytical pipeline to prioritize drugs based on biomarkers: their approach indicated CDK4/6 hyperactivation and BETs as druggable targets in OS, which were validated in the same mPDX cohort in vivo [71]. While mPDXs constitute living biobanks, embryonal models represent instead short-time hosts from which a limited amount of material is retrievable at the end of xenografting experiments. However, given their fidelity [176,177,178], embryonal models have peculiar advantages in drug-screening applications. Indeed, they enable middle-to-high throughput studies that are feasible in the context of an intact living system recapitulating human physiology and pharmacokinetics in a reasonable timeframe [165,170]. Moreover, drug administration can be performed by juxtaposition in ovo or by dissolution into ZF embryo water, simplifying handling. A streamlined experimental procedure employing only one drug has been employed to calculate the IC50 of vincristine in ovoCDX of established RMS cells [179], providing a proof-of-principle for the employment of the CAM model as a platform for drug screening. However, a drawback of the use of ovoCDX in drug screening is the current lack of automated high-throughput procedures, which are feasible in ZF. By analogy with ZeOncoTest [180], Grissenberger and colleagues exploited high-content images of xenografted fluorescent EWS cells to follow tumor growth in ZF. This approach permitted the screening of 16 compounds and their combinations in groups of 30–95 larvae over a 3-day period of time in two cell lines and related models with inducible knock-down of EWS::FLI1 [181]. The obtained results were validated in a mPDX, with a significant reduction of mammalian animal models, costs, and experimental time. Understandably, mPDXs are difficult to employ for large high-throughput drug screening due to high costs and cumbersome handling [174]. Once the drug has been selected, mCDXs and, lately, mPDXs, represent the most effective preclinical models for the evaluation of drug efficacy. mCDXs have represented the principal modality of preclinical drug testing for decades—their employment is still pivotal for near-patient [182], drug-resistant [183], and gene-edited [184] cells, rarer disease entities (SS [185,186], Epithelioid sarcoma [187], MFS [188]), and they often represent a preliminary step to mPDXs. In recent decades, preclinical drug evaluation has moved to mPDXs as the most trustworthy model. Accordingly, all the most promising molecules currently in clinical trials for sarcomas have been tested on mPDX (Table 5). Moreover, the efficacy of the inhibitors of (i) proteasome (VLX1570) [189], H3K27ac (GSK-J4), CDK7-mediated transcriptional process (THZ1) [190], mevalonate pathway (atorvastatin) [191] in EWS, (ii) ATR [192] and the combination of Aurora Kinase A *plus* navitoclax [193] in RMS, (iii) irinotecan and axitinib in LPS [194], and (iv) ATR in SS [195] was validated in mPDX after drug screening. Most importantly, the creation of mPDX models of ultra-rare sarcomas permitted the evaluation of trabectedin, eribulin, and various combinations of chemotherapeutics in solitary fibrous tumors [196], doxorubicin, eribulin, temozolomide *plus* irinotecan, and the targeted drugs palbociclib and linsitinib in an orthotopic mPDX of FUS-ERG EWS [197,198,199], as well as the combination of c-MET, EZH2, mTOR inhibitors, and ADM *plus* trametinib in epithelioid sarcoma [200,201]. However, preclinical testing on mPDX does not warrant success in clinical evaluation [9]. Indeed, the robustness of results is restrained by the limited number of experimental groups (<10 mice/group) and disease entities of origin. Some notable examples of drugs which failed to enter clinical praxis after exceptional preclinical results are represented by eribulin-, olaratumab-, and Akt-directed therapies. In some cases, after an unsuccessful clinical trial, researchers returned to their preclinical models to identify possible biomarkers of response. Low Bcl-2 expression was identified as a possible biomarker of response to nab-paclitaxel, and its inhibition via venetoclax was found to be synergistic [202], while low SPARC expression was associated with low drug retention and inefficacy [203]. Several papers go through the entire path of drug discovery, especially in the context of ultra-rare tumors. This approach permitted the identification, screening, and validation of anti-cancer drugs in the context of (i) CIC-DUX4 Ewing-like sarcomas [204], (ii) desmoplastic round cell sarcoma [205,206,207,208], (iii) mesenchymal chondrosarcoma [209], (iv) GCTB [210], and (v) clear cell sarcoma [211].

A particular mention should be paid to the extremely reduced number of cells needed for zCDX (<10^3^), which makes it an attractive and suitable system to xenograft near-patient cells. In this context, the efficacy of bone-targeted therapy (denosumab), TK inhibitors (lenvatinib), and their combination was evaluated on the zCDX of primary cells of desmoplastic fibroma [212], while trabectedin was evaluated on patient-derived primary cultures of UPS [213].

**Table 5:** Open clinical trials with matching preclinical research on MSKT. The table reports details of recent clinical trials in MSKT on the left, while on the right the details of the matched preclinical research on mPDX are reported. n.d., not disclosed.

Clinical Trial			Preclinical Research		
NCT Number	Study Title	Conditions	Mice/Group	N° mPDX/ Trial	Refs.
NCT03838744	Randomized Trial in Advanced, Metastatic, or Unresectable Soft Tissue Sarcoma After Failure of Standard Treatments	Advanced Soft Tissue Sarcoma	5	2	[214]
NCT04076579	Trabectedin in Combination With Olaparib in Advanced Unresectable or Metastatic Sarcoma	Sarcoma; Sarcoma Metastatic			[214]
NCT03936465	Study of the Bromodomain (BRD) and Extra-Terminal Domain (BET) Inhibitors BMS-986158 and BMS-986378 in Pediatric Cancer	Pediatric tumors and Lymphoma	7	1	[215]
NCT04095221	A Study of the Drugs Prexasertib, Irinotecan, and Temozolomide in People With Desmoplastic Small Round Cell Tumor and Rhabdomyosarcoma	Rhabdomyosarcoma	6	6	[216]
NCT03709680	Study Of Palbociclib Combined With Chemotherapy In Pediatric Patients With Recurrent/Refractory Solid Tumors	Pediatric solid tumors	6	6	[199]
NCT04238819	A Study of Abemaciclib (LY2835219) in Combination With Other Anti-Cancer Treatments in Children and Young Adult Participants With Solid Tumors, Including Neuroblastoma	Relapsed Solid Tumor; Refractory Solid Tumor	3	1	[217]
NCT05071209	Elimusertib for the Treatment of Relapsed or Refractory Solid Tumors	Recurrent/Refractory Alveolar Rhabdomyosarcoma; Recurrent/Refactory Ewing Sarcoma;	5–6	2	[218]
NCT04067115	SARC037: A Phase I/II Study to Evaluate the Safety of Trabectedin in Combination With Irinotecan in Ewing Sarcoma Patients	Ewing Sarcoma	6	1	[208]
NCT04537715	Study to Describe the Interaction Between Tazemetostat and Itraconazole and Between Tazemetostat and Rifampicin in Participants With Advanced Cancer	Epitelioid Sarcoma; Synovial Sarcoma	n.d.	6	[219]
NCT03793361	Phase II Study of Regorafenib as Maintenance Therapy	Metastatic Soft Tissue Sarcoma	10	7	[220]
NCT05515575	A Study of Niraparib in People With Soft Tissue Sarcoma Who Have Changes in Their Tumor DNA	Sarcoma, Soft Tissue	7	1	[221]
NCT05218499	Brightline-1: A Study to Compare Brigimadlin (BI 907828) With Doxorubicin in People With a Type of Cancer Called Dedifferentiated Liposarcoma	Dedifferentiated Liposarcoma	6	2	[222]
NCT04742959	Study of TT-00420 (Tinengotinib) Tablet as Monotherapy and Combination Therapy in Patients With Advanced Solid Tumors	Advanced solid tumors	7–8	3	[203]
NCT02095132	Adavosertib and Irinotecan Hydrochloride in Treating Younger Patients With Relapsed or Refractory Solid Tumors	Relapsed or Refractory Solid Tumors	10	4	[223]
NCT04950075	Study of INBRX-109 in Conventional Chondrosarcoma (ChonDRAgon)	Conventional Chondrosarcoma	8	2	[224]
NCT03718091	M6620 (VX-970) in Selected Solid Tumors	Advanced solid tumor	6	1	[225]
NCT03810976	A Study of Eribulin With Gemcitabine in Patients With Advanced Liposarcoma or Leiomyosarcoma	Liposarcoma or Leiomyosarcoma	3–4	3	[226]
NCT04794127	Study of Trabectedin in Combination With Pioglitazone in Patients Myxoid Liposarcomas With Stable Disease After T Alone (TRABEPIO)	Myxoid Liposarcomas	10	3	[227]

## 7. Combinatorial Approaches

The combination of different living hosts for xenografts represents a win-win approach which exploits the peculiar traits of each model for specific scientific questions, providing on the one hand the possibility to dissect disease mechanisms with higher robustness, and on the other a more comprehensive insight at the organismal level [228]. Below, some examples of this virtuous approach are reported.

Pignochino et al. demonstrated the efficacy of sorafenib in OS stepwise: the anti-angiogenic action was assessed on naked CAM exposed to the drug itself or to the conditioned medium of cells treated with the drug. As a second instance, the efficacy of sorafenib in restraining the tumorigenic and metastatic ability of OS cells was evaluated via heterotopic mCDX [229]. Analogously, in both ovoCDX and mCDX, and in a mPDX model, the treatment with verteporfin significantly slowed tumor growth. In this case, models were used stepwise from the most time-effective and scalable (ovoCDX) to the most complex, time-consuming, yet near-patient (mPDX), yielding consistent results [127]. Tome and colleagues seeded 143B-LM-RFP OS cells in ovo—the derived tumor masses were then transplanted into several other eggs and treated with echistatin. This protocol allowed the drug to be tested in an established tumor. Subsequently, the efficacy of echistatin diminishing the tumorigenic and metastatic potential of the same cells was demonstrated in mCDXs, in combination with doxorubicin [230]. Analogously, the pro-angiogenic role of soluble BDGF in CHS was firstly assessed in the absence of cells both in ovo and in mice, thereafter, an mCDX of BDGF knock-down cells confirmed the finding at the organismal level [231]. Additionally, the role of IGFB6 on angiogenesis was firstly elucidated injecting its mRNA in the one-stage *flk1*-GFP embryo in a physiological context—the anti-angiogenic effect was later confirmed in a disease-related context with the injection of RMS cells in mice [232]. Concerning studies in the metastatic setting, Adane and colleagues, after intensive molecular investigations, found confirmation of the anti-tumorigenic role of STAG2 firstly in ZF, where its loss was associated with a higher percentage of embryos with migrated cells, and later in mice, where the injection of the same STAG2-KO cells yielded more lung metastasis with respect to control cells [233].

Generally, drug screening studies proceed stepwise. For example, after identifying active compounds targeting FAK and Aurora Kinase B in EWS via a high-throughput chemical and genetic screening, Wang et al. preliminarily assessed the pharmacokinetic properties and efficacy of zCDX prior to mCDX and mPDX [234]. Remarkably, since all the hosts were characterized by immunodeficiency, the interspecific transfer of cells or tissues from one to another was feasible. Indeed, Yan and colleagues performed high-throughput drug screening on near-patient material in vitro—the most promising drugs were selected for further evaluation and performed on zCDX of dissociated mPDX tumors of two rhabdoid sarcomas [235].

## 8. Discussion

Survival rates for most MSKT have not improved since the 1980s, when chemotherapy was introduced. This stagnation reflects, on the one hand, a lack of understanding of the biological mechanisms underlying the sarcomagenesis and metastasis processes, and on the other a lack of reliable models to identify effective tumor targets and to testing new drugs preclinically [7,9].

Historically, most of the preclinical research has involved xenografts of cells or tumor fragments in adult rodents, especially mice. Recently, researchers have turned their attention to embryonal models such as chick embryo and zebrafish, which not only represent time- and cost-effective alternative hosts, but most importantly minimize pain, environmental distress and, hence, ethical concerns related to animal experimentation.

With regard to basic research, mCDX involving established gene-edited or drug-resistant cells have permitted the elucidation of the role of tens of genes modifying their tumorigenic or metastatic power (Table 3 and Table 4). In this experimental setting, the integration of fast-developing embryonal models has allowed, on the one hand, the employment of the same transient strategies used in vitro in the in vivo setting and, on the other hand, has raised the possibility of evaluating the phenotype of gene-edited near-patient cells. Thus, embryonal models contribute to time-effectiveness, consistency in methodology, and translatability of results. Murine models offer the unique possibility of studying all the different phases of the natural history of tumors with incomparable reliability, due to the close homology with humans at many levels [7,9,115], albeit blindly. Conversely, embryonal models permit the dissection and visualization of the dynamic interactions between cancer cells, vessels, and the host’s organs, even at single-cell level. Particularly, embryonal models allow the visualization and quantification of sprouting vessels, and the dissemination of tumor cells upon xenografting tumor fragments or cells [144,164,170,177,236]. Notwithstanding these features, researchers must keep in mind that embryos are miniaturized immunodeficient organisms under development, with high levels of specific growth factors, and thus lack target organs and time to model a full-blown metastasis.

Concerning translational research, preclinical trials have successfully relied on mCDX until mPDX took root at the beginning of the century. This practice allowed the expansion and biobanking of patient-derived material, which is particularly relevant in the case of rare and heterogeneous tumors, such as bone and soft tissue sarcomas [9]. mPDXs are acknowledged as the most effective preclinical model for recapitulating intrinsic cancer biology and architecture, and studying drug response and resistance [9]. Preclinical trials taking advantage of mPDXs are relevant throughout the whole course of drug development, including the phases in clinical experimentation. This is important in the context of rare tumors, like sarcomas, for which most clinical studies include patients of all sarcoma histotypes and in small case series. For these reasons, the use of PDX models represents a precious method in preclinical cancer research. Trabectedin is a paradigm of the usefulness of mPDX in the drug development process. This drug was approved in Europe in 2007 for the treatment of advanced STS after the failure of anthracycline and ifosfamide [237]. In 2015, the FDA endorsed its use for the treatment of previously treated advanced metastatic liposarcomas and leyomiosarcomas [238]. mPDXs were used to test trabectedin’s efficacy on sarcomas [239], and recently they were leveraged for the investigation of the molecular mechanisms of actions and of resistance [69]. These findings may give rise to innovative therapeutic combinatorial strategies enhancing the trabectedin effects. Another example of mPDXs’ utility in the management of trabectedin is represented by a recent paper by Zuco et al. studying new therapeutic approaches for desmoplastic small round cell tumor (DSRCT). The authors found that the combination of trabectedin with irinotecan resulted in complete responses in mice trials. These findings strongly support further investigations of this combination in clinical studies on relapsing DSRCT, moreover, these results can also be translated for the development of Lurbinectidin, a novel synthetic agent derived from trabectedin with a similar mechanism of action and that is currently under investigation in combination with irinotecan in sarcomas (NCT02611024) [208]. Nevertheless, preclinical testing on mPDX does not warrant success in clinical evaluation. In this scenario, the introduction of embryonal models in the screening phase of drug development, upon their validation as New Approach Methodologies (NAM), could provide mid-to-high throughput systems in the context of an intact living organism recapitulating human physiology and pharmacokinetics in a reasonable timeframe [165,170]. Finally, the little material required for zCDX and the short experimental time in both embryonal systems makes them appealing as “avatar models” or “mirror models” in a time-frame congruent with clinical decision making [212,213].

Currently, thanks to their predictive value, xenografts are used in co-clinical trials in which the xenograft is treated in parallel with the corresponding patient. This approach facilitates the prioritization of optimal treatments, simplifies rapid classification of responders, identifies biomarkers, and detects mechanisms of resistance [240]. This format of trial has also been conducted for sarcomas [241], with excellent results of concordance between drug response in mice and in corresponding patients. Moreover, not only are mPDXs used in co-clinical trials, but zPDXs are also, although not for sarcomas and instead for other solid tumors [51].

Consisting of multiple cell types and an extracellular matrix, tumors engage in complex interactions with surrounding tissues and the entire body. As a result, they are increasingly perceived as organs [242], the complexity of which cannot be modeled by a single system, neither in vitro nor in vivo. Therefore, while the choice of the xenograft host should align with the experimental question, the combination of different xenograft models based on cells and patient-derived material and the integration of omic, in vitro, and in vivo data could provide robustness to preclinical results [228]. Moreover, the adoption of stepwise approaches envisioning embryonal systems as a bridge between in vitro and in vivo murine experiments could corroborate the first, while reducing the number of more complex animals needed, in compliance with the 3R principles.

## 9. Conclusions and Future Perspectives

In the present work, we reviewed the literature reporting xenografts of human musculoskeletal sarcomas in mouse, zebrafish larvae, and chick embryos. The categorization of papers based on the underlying biological question allowed us to highlight the intrinsic and technical peculiarities of patient-derived and cell-derived xenografts across the phases of tumor natural history and drug development. Embryonal models emerged as potential tools to corroborate results obtained in gene-edited systems in vitro, using the same transient approaches, thereby increasing robustness and reducing experimental times. Embryonal models are promising systems for the study and visualization of stepwise, dynamic, and interactive processes such as angiogenesis and metastatization, thanks to the ease of imaging and reduced experimental windows. On the other hand, murine xenografts allow the study of overt metastatization and the creation of living biobanks of sarcomas. Thanks to their fidelity, mPDXs represent the gold standard for drug development, which could benefit from the introduction of high-throughput embryonal systems in the screening phase.

Overall, the application of embryonal systems in sarcomas is still limited. Yet, the combination of different hosts for xenografts in stepwise approaches involving near-patient material promises to provide reliable high-throughput and highly informative models, boosting both basic and translational research in respect of the 3R principles

## Data Availability

No new data were created or analyzed in this study. Data sharing is not applicable to this article.

## Acronyms

Adm	Adrenomedullin
Akt	AKT Serine/Threonine Kinase 1
AI	Artificial intelligence
ALU	stretch of DNA characterized by the action of endonuclease from *Arthrobacter luteus (Alu)*
Atr	ATR Serine/Threonine Kinase
Bcl-2	B-Cell Leukemia/Lymphoma 2 Protein.
Bdgf	Brain-Derived Nerve Growth Factor
Bets	Bromodomain Extraterminal Domain Proteins
Bmp-2	Bone Morphogenetic Protein 2
Cav1	Caveolin 1
Cdk	Cyclin-Dependent Kinase
Cdkn1c/2a	Cyclin Dependent Kinase Inhibitor 1c/2a
Chek1	Checkpoint Kinase 1
Cic-Dux4	Fusion; Capicua Transcriptional Repressor- Double Homeobox 4
CMET	Hepatocyte Growth Factor Receptor
Csf1r	Colony-Stimulating Factor-1
Cxcr4	C-X-C Chemokine Receptor Type 4
Ews::Fli1	Fusion; EWS RNA Binding Protein 1:: Fli-1 Proto-Oncogene, ETS Transcription Factor
Ext1/2	Exostosin Glycosyltransferase 1
Eya3	Eya Transcriptional Coactivator and Phosphatase 3
Ezh2	Enhancer of Zeste 2 Polycomb Repressive Complex 2 Subunit
Fak	Focal Adhesion Kinase
FDA	Food and Drug Administration
Fgd1	Fyve, Rhogef And Ph Domain Containing 1
Fli1	Friend Leukemia Integration 1 Transcription Factor
Foxp1	Forkhead Box Protein P1
Fus-Chop	Fusion Protein; Fus RNA Binding Protein- DNA Damage Inducible Transcript 3
Fus-Erg	Fusion; FUS RNA Binding Protein- ETS Transcription Factor ERG
Gfra1	Gdnf Family Receptor Alpha-1
Gli	GLI Family Zinc Finger 1
Gαi3	Guanine Nucleotide-Binding Protein G(I) Subunit Alpha-3
H3k27ac	Acetylation of Histon3 in lysine (k) 27
Hdac	Histone Deacetylases
Hes1	Hes Family Bhlh Transcription Factor 1
IC50	Half Maximal Inhibitory Concentration
IDH1/2	Isocitrate Dehydrogenase (NADP(+)) 1/2
Igf2bp3	Insulin Like Growth Factor 2 mRNA Binding Protein 3
Igfb6	Insulin Like Growth Factor Binding Protein 6
Igfbp5	Insulin-Like Growth Factor-Binding Protein 5
IHC	immunohistochemistry
Itga5	Integrin Subunit Alpha 5
Kif18b	Kinesin Family Member 18b
Klf15	Klf Transcription Factor 15
Lpar1	Lysophosphatidic Acid Receptor 1
Mdm2	MDM2 Proto-Oncogene
Mig-7	Migration Inducting Gene 7
mTOR	Mammalian Target of Rapamycin
Ng6x	Nasopharyngeal Carcinoma-Associated Gene 6
Panx1	Pannexin 1
Pax(n)::FoxoA1	Fusion; Paired Box (n):: Forkhead Box O1
PDGRF-Β	Platelet-Derived Growth Factor Receptor Beta
Pdpn	Podoplanin
Plk2	Polo Like Kinase 2
Porcn	Porcupine O-Acyltransferase
Pten	Phosphatase And Tensin Homolog
qPCR	Quantitative Polyerase Chain Reaction
Rab22a::Neof1	Fusion Protein; Ras-Related Protein Rab-22A
Ras-PI3K	RAS Proto-Oncogene, GTPase-Phosphatidylinositol-4,5-Bisphosphate 3-Kinase
Rb1	RB Transcriptional Corepressor 1
Ripk1	Receptor Interacting Serine/Threonine Kinase 1
Rock2	Rho Associated Coiled-Coil Containing Protein Kinase 2
Ron	Macrophage Stimulating 1 Receptor
S1pr3	Sphingosine-1-Phosphate Receptor 3
Sh Gαi3	Short Hairpin Guanine Nucleotide-Binding Protein G(I) Subunit Alpha-3
Sh Foxp1	Short Hairpin Forkhead Box P1
Sirt1/2	Sirtuin 1/2
Smo::	Smoothened, Frizzled Class Receptor
Sox6	Sry-Box Transcription Factor 6
Syt::Ssx1/2/4	SS18 Subunit Of BAF Chromatin Remodeling Complex:: SSX Family Member 1/2/4
Sparc	Secreted Protein Acidic and Cysteine Rich
Stag2	Stag2 Cohesin Complex Component
Stat3	Signal Transducer and Activator of Transcription 3
Tap73	Tumor Protein P73
Taz	Transcriptional Coactivator with Pdz-Binding Motif
Tcf4	Transcription Factor 4
Tead	Tea Domain Transcription Factor
Timm13	Translocase Of Inner Mitochondrial Membrane 13
Tp53	Tumor Protein P53
Tk	Tyrosine Kinases
Trim7	Tripartite Motif Containing 7
Wnt	Wingless/Integrated
Yap1	Yes-Associated Protein 1
Yhr	Yaps127a-Htert-Hrasg12v
Yhv	Yaps127a-Htert-Vector
Zyx	Zyxin
